# Case Report: Low Bone and Normal Lean Mass in Adolescents With Complete Androgen Insensitivity Syndrome

**DOI:** 10.3389/fendo.2021.727131

**Published:** 2021-08-30

**Authors:** Aaron Misakian, Michelle McLoughlin, Louisa C. Pyle, Thomas F. Kolon, Andrea Kelly, Maria G. Vogiatzi

**Affiliations:** ^1^Department of Pediatrics, Children’s Hospital of Philadelphia, Philadelphia, PA, United States; ^2^Division of Urology, Children’s Hospital of Philadelphia, Philadelphia, PA, United States

**Keywords:** bone mineral density, complete androgen insensitivity syndrome, gonadectomy, lean mass, osteoporosis

## Abstract

**Introduction:**

Osteopenia and osteoporosis have been reported in adults with Complete Androgen Insensitivity Syndrome (CAIS). Little is known about changes in bone mineral density (BMD) in adolescents with CAIS and whether it is affected by early gonadectomy. Body composition data have not been reported.

**Methods:**

Single-center, retrospective study of CAIS adolescents who underwent dual-energy x-ray absorptiometry (DXA) (Hologic, Horizon A). Body composition is presented as lean and fat mass indices (LMI, FMI). Z-scores for lumbar spine areal BMD (LBMD), total body less head (TBLH), bone mineral content (BMC), LMI, and FMI were calculated using female normative data. Results are expressed as median and min, max.

**Results:**

Six females with genetically confirmed CAIS were identified—one with intact gonads and five with history of gonadectomy at 2–11 months. In the subject with intact gonads, LBMD-Z and TBLH BMC-Z were −1.56 and −1.26, respectively, at age 16 years. Among those with gonadectomy, LBMD-Z was −1.8 (−3.59 to 0.49) at age 15.6 years (12–16.8) and decreased in all three subjects who had longitudinal follow-up despite hormone replacement therapy (HRT). Adherence to HRT was intermittent. LMI-Z and FMI-Z were 0.1 (−1.39 to 0.7) and 1.0 (0.22 to 1.49), respectively.

**Conclusions:**

These limited data indicate that adolescents with CAIS have bone mass deficit. Further studies are needed to understand the extent of BMD abnormalities and the effect of gonadectomy, especially early in childhood, and to establish the optimal HRT regimen for bone accrual. Data on lean mass are reassuring.

## Introduction

Complete androgen insensitivity syndrome (CAIS) (OMIM# 300068) is an X-linked recessive disorder characterized by mutations of the androgen receptor (AR) that render the receptor completely non-functional (1, 2) and occurs in 1:20,000 to 90,000 neonates (2). In the fetus affected by CAIS, the fully functional SRY protein on the Y chromosome permits male gonad development, normal secretion of testosterone and anti-Mullerian hormone, and ultimately regression of the Mullerian structures destined to be the uterus and fallopian tubes (2). However, the fetus cannot properly respond to androgens (3) and virilization of external genitalia is absent (4). Male gonads remain intact with retention of testis in the abdomen or inguinal area (5). Thus, while CAIS individuals are genetically XY, they appear phenotypically female and are usually raised and legally recognized as female (1).

Treatment for CAIS is multifactorial and includes vaginal enlargement, genetic counseling, and psychosocial support. Gonadectomy for prevention of germ cell tumor development and hormone replacement therapy (HRT), typically estrogen, are usually recommended although the timing of gonadectomy is controversial (6). Because testosterone is metabolized into 5a-dihydrotestosterone (5-DHT) *via* 5a-reductase-2 or into 17B-estradiol *via* P450 aromatase, adolescents with CAIS and intact gonads are able to achieve the expected effects of puberty including typical breast development, widening of the hips, redistribution of fat, growth acceleration, and bone accrual *via* 17B-estradiol, while they lack axillary and pubic hair development (6).

Testosterone and estrogen play critical roles in bone mineral accrual during adolescence and young adulthood, a life stage during which peak bone mass is achieved and risk of osteoporosis and fracture at least partly established (7). To date, limited data explore changes in bone mineral density (BMD) in CAIS adolescents (8–10). Instead, most publications focus on bone health in adult CAIS women (11–20). Understanding the effects of early *vs.* late gonadectomy, the impact of hypogonadism, and its treatment during adolescence is important as peak bone mass and accrual occur during this time (7). Furthermore, body composition can influence both skeletal health and cardiometabolic risk factors (21–23), and particularly relevant in CAIS is the impact of hypogonadism and gonadal hormones on lean mass and fat distribution (24–27). To better understand such potential alterations in adolescents with CAIS, we describe BMD, body composition, and changes in these outcomes in a series of patients treated with HRT.

## Methods

We performed a retrospective cohort study of all CAIS females (age <20 years) who had follow-up by the Division of Endocrinology at our institution between July 1, 2015, and December 30, 2020, and underwent bone mass (lumbar area and whole body) and body composition measurements by dual-energy x-ray absorptiometry (DXA) (Hologic, Horizon A). Data extracted from medical charts included age, height, weight, body mass index [BMI], tanner stage, presence of gonads, age at gonadectomy, medications including type of HRT and age at treatment initiation, comorbidities, and laboratory or radiographic evaluation (serum estradiol concentrations, DXA, bone age results). Data collection was approved by the Institutional Review Board.

Z-scores for lumbar spine areal BMD (LBMD) and total body less head (TBLH) bone mineral content (BMC) were calculated using female normative data. Body composition results are presented as lean body mass and fat mass indices (LMI, FMI), which were calculated as kg of lean or fat mass respectively/m^2^. Height, weight, body mass index (BMI), LMI, and FMI Z-scores were calculated using female normative data (28). Overweight and obesity were defined using the Centers for Disease Control (CDC) classification (29). Height and weight Z-scores were calculated using the CDC growth charts. Results are expressed as median and range (i.e., min-max). Z-scores within 1SD from population mean are considered normal.

### Subjects

**Figure 1:**
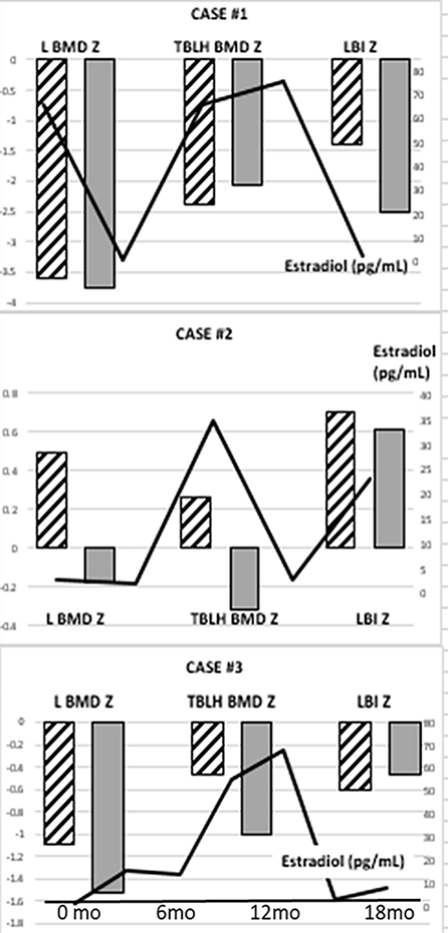
Lumbar (L) and Total Body Less Head (TBLH) BMD Z-scores and LMI-Z in three adolescents with CAIS, history of early gonadectomy, and longitudinal DXA measurements. Hatched columns represent the results of the initial DXA study and gray columns represent the follow-up DXA results. Estradiol levels throughout the time of follow-up are shown as a solid line.

We identified six subjects with CAIS—one had intact gonads and five had history of gonadectomy early in life. Gonadectomy was performed between 2 and 11 months (median age 5.6 months). HRT was initiated at an age of 12 years (range 10.5–14.2) using estradiol as either a transdermal patch or oral pill. HRT was started at low doses but increased to achieve appropriate growth and acquisition of secondary sexual characteristics with a max of 0.1 mg/day (patch), 1 mg orally once daily (Estrace), or 0.9 mg orally once daily (Premarin) (**Table 1**). Induction of puberty was late in Subjects 4 and 5 because families had not fully disclosed the diagnosis and patients were lost to follow-up after gonadectomy before eventually returning to care. Compliance with HRT ranged from poor to good as evidenced by serum estradiol levels (**Figure 1**), which were obtained in those treated with transdermal estrogen for clinical surveillance. Height, weight, and BMI Z-scores are presented in **Table 1**. One subject (patient 2) was obese, and subjects 4 and 5 were overweight. Subjects 1, 3, and 6 had reached adult height at the time of the first DXA. The rest of the subjects were still growing when the first DXA was performed. Subject 2 had a growth rate of 5 cm/year and a bone age that was concomitant to chronologic age (bone age 12 years, chronologic age 12 years 1 month). Subject 4 had reached a near-adult height growing at 2.5 cm/year. Her bone age was 14 years at the chronologic age of 16 years 6 months. Subject 5 was growing at a rate of 7.2 cm/year. Her bone age was 12 years at the chronologic age of 13 years 8 months. Subjects 1, 2, 3, and 5 received vitamin D supplementation to optimize bone health. Subjects 1–3 achieved serum 25-OH-vitamin D concentrations >30 ng/ml. The 25OHD has not yet been obtained for subject 5. Subjects 4 and 6 have not started vitamin D. Information on calcium intake is not available.

**Table 1:** Baseline characteristics, type/timing of HRT, and results of first DXA scans for all six cases.

Subjects	Age at GND(mo)	Age HRT started(yr, mo)	Age 1^st^ DXA(yr, mo)	Duration HRT at time of 1^st^ DXA(yr, mo)	Weight Z	Height Z	BMIZ	Lumbar BMDZ	TBLHBMCZ	LBMI Z	FMI Z	HRT	Compliance
**1**	2	10, 11	15, 6	4, 7	+0.04	-0.12^A^	+0.1	-3.59	-2.39	-1.39	+0.22	E2 Patch: 14 mcg/24 h for 1 yr → 37.5 mcg/24 h for 1 yr → 50 mcg/24 h for 1 yr → 100 mcg/24 h for 1 yr (current therapy)	Intermittent
**2**	6	10, 5	12, 1	1, 8	+1.42	-0.03	+1.65	+0.49	+0.26	0.7	+1.03	E2 Patch: 14 mcg/24 h for 1 yr → 37.5 mcg/24 h for 6 mos → 50 mcg/24 h for 6 mos → 75 mcg/24 h for 6 mos → 100 mcg/24 h for 1 yr, 5 mos → Estrace 1 mg po daily (current therapy)	Poor
3*	11	11, 11	16, 0	4, 1	+1.05	+0.98^A^	+0.75	-1.1	-0.46	-0.6	+0.46	E2 Patch: 14 mcg/24 h for 1 yr → 50 mcg/24 h for 8 mos → 75 mcg/24 h for 7 mos → 100 mcg/24 h for 1 yr → stopped all estrogen therapy for 3 mos → Premarin 0.9 mg for 8 mos → E2 Patch: 50 mcg/24 h for 2 yrs → increased to 75 mcg/24 h (current therapy)	Intermittent
**4**	3	14, 2	16, 8	2, 6	+1.69	-0.17	+1.77	-1.8	-0.44	0.49	+1.49	Estrace: 0.25 mg daily for 3 mos → 0.50 mg daily for 7 mos → 0.75 mg daily for 1.4 yrs → 1.0 mg daily (current therapy)	Good
**5**	6	12, 10	13, 6	0, 8	+1.39	-0.27	+1.6	-2.65	-1.51	0.1	+1.29	E2 patch: 14 mcg/24 h for 7 mos → 25 mcg/24 h for 5 mos → 37.5 mcg/24 h for 16 mos → 50 mcg/24 h (current therapy)	Intermittent
**6**	N/A	N/A	16	N/A	-0.34	+0.83^A^	-0.96	-1.52	-1.26	-0.6	-1.52	N/A	N/A

GND, gonadectomy; HRT, hormone replacement therapy; Z, Z-score; BMD, bone mineral density; TBLH, total body less head; TBLH BMC, total body less head bone mineral content; LBMI, lean body mass index; FMI, fat mass index; MS, Menostar transdermal patch (changed once weekly); VD, Vivelle-Dot transdermal patch (changed twice weekly); CL, Climara transdermal patch (changed once weekly). N/A, not applicable. *Subject 3 has Sickle Cell Anemia (Type SS). ^A^Subject has reached adult height.

#### DXA Results

##### Subjects With History of Gonadectomy

DXA scans were obtained at a median age of 15.6 years (range 12–16.8) after receiving HRT for a period of 2.5 years (range 0.8–4.6). Patients 1–3 had serial DXAs over time to assess response to HRT.

Median age-adjusted lumbar areal BMD-Z (LBMD-Z) was −1.8 (range −3.59 to 0.49) at the initial screen (**Table 1**) with two subjects (Cases 1, 5) having Z-scores < −2. The data from longitudinal follow-up are presented in **Figure 1** and show a decline in LBMD-Z over 2.8 years (range 1.7–3) of observation. Total body less head (TBLH) BMC was less affected at baseline (median Z = −0.46, range −2.39 to 0.26, **Table 1**); results varied during follow-up with subject 1 showing mild improvement. LMI-Z was normal in all subjects except 1 (Subject 1) with median LMI-Z +0.1 (range −1.39 to 0.7); results varied during follow-up. FMI-Z was elevated (greater than +1) in patients 2, 4, and 5 and normal in the rest of the subjects.

##### Subject With Intact Gonads

The adolescent (Case 6) was studied at age 16 years after undergoing spontaneous puberty. LBMD-Z and TBLH BMC Z-scores were −1.52 and −1.26, respectively. LMI-Z and FMI-Z were −0.6 and −1.52, respectively (**Table 1**).

## Discussion

Bone density data in children and adolescents with CAIS are sparse. In this report, we assessed BMD and body composition in a series of five adolescents with CAIS who underwent gonadectomy during infancy. We observed low lumbar BMD (Z < −1) in all but one adolescent at the time of the first DXA. Longitudinal follow-up in three subjects with intermittent compliance to estrogen replacement showed a decrease in lumbar BMD. Given our small numbers and the observational nature of this report, we cannot reach any firm conclusions about estrogen replacement and bone health in CAIS. However, our data raise concerns about a negative effect of poorly treated hypogonadism on the skeletal health of adolescents with CAIS. In this series, lean mass was grossly normal and fat mass was reflective of BMI. A single additional adolescent with intact gonads and spontaneous puberty also had low lumbar BMD and normal LMI-Z.

CAIS is a disease model that allows us to explore the role of androgens on the skeleton. Bone tissue is sexually dimorphic (30, 31). Estrogens limit periosteal bone expansion yet stimulate endosteal bone apposition in females, while androgens promote radial bone expansion in males (30). Both estrogen and testosterone play important roles in bone phenotype, and their combined effects lead to men generally having wider but not denser skeletons compared to women. Bone size in testicular feminized male rats is intermediate between male and female control animals (32). In pre-pubertal children with CAIS, alterations in bone mass are likely to reflect the role of androgens on the bone.

In adolescents and adults with CAIS, bone mass may be further affected by gonadectomy. Because of the risk for testicular germ cell tumors (GCTs), prophylactic gonadectomy has been recommended in CAIS patients although optimal timing for the procedure remains a topic of debate (6, 33, 34). Gonadectomy early in life at the time of diagnosis has been practiced for years. Since the risk for GCTs is low in children, gonadectomy after completion of spontaneous puberty *via* aromatization of endogenous testosterone has also been proposed by some groups (19, 33). More recently, some women with CAIS elect to defer gonadectomy to benefit from endogenous sex hormone secretion (6, 33). Once gonadectomy is performed, the individual becomes hypogonadal and is placed on estrogen replacement. Relevant to bone health, the ideal HRT regimen for bone accrual and maintenance is not well established. Changes in bone mass in CAIS, therefore, should be interpreted keeping in mind whether gonadectomy has been performed, when such procedure took place, as well as the type of estrogen replacement regimen and patient adherence.

BMD data in CAIS are more recently expressed using female normative data. Whether this approach is appropriate is uncertain since fracture data in CAIS are not available. As BMD serves as a predictor of fractures, understanding bone fragility and relationship with BMD in CAIS may shed light on the most appropriate way to assess bone mass and optimal HRT in this population.

Reduced bone mass has been reported in adults with CAIS (**Table 2**). The information derives from retrospective, descriptive studies over the last decade (11–20). As a common theme, bone mass was affected primarily in the spine with BMD T-scores falling in the osteopenic range for most studies (11–20). Most of the published studies include women who were studied years after gonadectomy (**Table 2**); thus, their DXA results reflect the impact of the disease itself and sex hormone replacement on the skeleton. Poor compliance with HRT has been shown to be associated with worsening BMD in one of these studies (13), while transdermal therapy was found to be superior to oral estradiol in optimizing bone health in a study by Gava et al. (20). Longitudinal follow-up after gonadectomy yielded variable DXA results with estrogen replacement (18, 19). The reason for this diversity in HRT response is not fully understood. Furthermore, the specific estrogen regimens are not clearly detailed in all studies and vary among reports, so identifying an optimal estrogen regimen for bone health in CAIS based on the current literature is challenging.

**Table 2:** BMD studies in adults with CAIS.

Reference	Gonadectomy				Intact Gonads			Comments
	N	Age at GND	Age at DXA	Lumbar BMD T	N	Age at DXA	Lumbar BMD	
Soule et al. (10)	4	15.8+12.9	32.7+10.4	-2.6+0.9	1	29	-1.5	
Mizumuma et al. (11)	N/A	N/A	N/A	N/A	2	19 & 28	-0.8 & -3.1	
Marcus et al. (12)	18	13.3 (<1-31)	41.7+9.1	-1.2+1.1	N/A	N/A	N/A	Poor compliance with E2 Rx was associated with greater lumbar BMD deficits. Six pts with CAIS had fractures. Lumbar BMD T in six additional PAIS gonadectomized adults was -0.54 (-1.95-1.3).
Sobel et al. (13)	10	23.4+8.6	35.2+14.3	-2.4+1.0	1	21	-3.0	Lumbar BMD T in six additional PAIS gonadectomized adults was -1.9+0.95.
Danilovic et al. (14)	3	16, 15.2, & 27.6	22, 25, & 24.3	-1.4, -1.65, & 0.4	2	24 & 21	-1.6 & -2.6	
Han et al. (15)	46	15.9+7.3	32.2+10.7	-1.29+1.2	N/A	N/A	N/A	Lumbar BMD was the same as in 18 46XY adults with GD and 25 46XX GD.
Taes et al. (16)	1	15	31	-3.4	N/A	N/A	N/A	After gonadectomy, E2 replacement resulted in a decreased endosteal circumference, increased cortical thickness and area, but unchanged periosteal circumference.
Bertelloni et al. (17)	43	NR	NR	Mean -1.9	10	NR	Mean -0.7	Fracture: n = 1/43 in the gonadectomized group only.
King et al. (18)	104	14.8 (13-16.5)	33.8 (31.4-36.2)	-1.34 (-1.55 to -1.33)	12	25.1* (18.3-52.3)	-1.2 (-4.2-1.0)	
Gava et al. (20)	32, 32 controls	12.3+7.9 (0-24)	34.5+10.4	-1.95+0.94	N/A	N/A	N/A	Transdermal estrogens were associated with better TB BMD. No subject had fractures.

GND, gonadectomy; NR, not reported; T, T-score; TB, total body; GD, gonadal dysgenesis; N/A, Not Available.

*refers to the age of gonadectomy with DXA performed shortly before gonadectomy.

Maintaining gonads after spontaneous puberty and until later in adult life has been proposed as a strategy to preserve bone health (6, 19). Current studies (**Table 2**) include a small group of subjects, mostly in their 20s and 30s with intact gonads. Although sample sizes are small to draw firm conclusions, collectively the data support a small BMD deficit in the lumbar spine. Comparisons between individuals with and without gonadectomy are provided in two studies; unfortunately, the results are conflicting. Bertelloni et al. observed significantly lower BMD-Z at the lumbar and femoral necks among those who underwent gonadectomy compared to those with intact gonads (18). In contrast, the study by King et al. showed that lumbar BMD-Z were similar in both gonadectomized and non-gonadectomized individuals and did not decrease after gonadectomy (19). Perhaps differences in estrogen replacement can explain the variable outcomes on bone health.

The pediatric experience using DXA to assess bone mass in CAIS is counted to less than 20 subjects (**Table 3**). The larger series of 10 children (seven with gonadectomy and three without) is dated back to 1998 and carries the inherent limitation of lack of large normative data to calculate BMD-Z (9). Nonetheless, in this series lumbar BMD-Z was reduced compared to controls. Three more recent cases of adolescents without gonadectomy observed a lumbar BMD-Z that ranged from −2.9 to +0.9 (10, 11, 14). Collectively, the data in adolescents and women with preserved gonads support some degree of bone mass deficit in CAIS, which can reflect the lack of androgen action on the growing skeleton. It is also possible that the endogenous estrogen concentrations, which are derived from testosterone conversion, are sufficient for breast development and growth but inadequate for optimal bone accrual. A bone mass deficit was also observed in our series. The variability in estrogen dose, regimens, and adherence and differences in body weight and fat mass may have contributed to our results. Given these limitations and the small sample size, we cannot delineate the individual effects of CAIS itself, surgical intervention, and adherence to estrogen therapy. Finally, DXA Z-scores may have been underestimated in our growing subjects with a delayed bone age (35, 36).

**Table 3:** BMD reports in adolescents (<18 years) with CAIS.

Reference	Gonadectomy				Intact Gonads		
	N	Age at GND	Age at DXA	Lumbar BMD Z	N	Age at DXA	Lumbar BMD
Munoz Torres et al. (7)	N/A	N/A	N/A	N/A	1	17	-4.1
Bertelloni et al. (8)	7; 15 controls	15.4+1.8	17.7+2.2	-2.5+0.8	3	4,11 & 16	-2.9+1.1
Marcus et al. (12)	2	<2.5	14 & 11	-0.1 & -1.43	2	14 & 12	+0.0 & +0.9
Sobel et al. (13)	N/A	N/A	N/A	N/A	1	17	-2.9
Chin et al. (9)	N/A	N/A	N/A	N/A	1	15	-0.6

GND, gonadectomy; N/A, Not Available.

Our series provides the first lean mass data using DXA in CAIS. Fat mass has been reported normal or increased in a previous report (20) and was elevated (FMI-Z above 1 SD) in three subjects in this series. Both lean and fat mass were recently linked to bone mass, insulin sensitivity, and cardiometabolic health (21–23). Potential deficits may have an adverse impact in all these health parameters. Testosterone, acting through the androgen receptor, stimulates protein synthesis and hypertrophy of muscle fibers (26), while suppression of endogenous testosterone production in young men results in decreased protein synthesis and muscle strength and in increased adiposity (27). One would predict, therefore, a decrease a lean mass in CAIS. In this series, however, we observed normal lean mass in all but one subject. Lean mass was calculated using female normative data, and whether this is the most appropriate assessment in CAIS is uncertain. Our subject numbers are small, and potential differences in lean mass in CAIS compared to the general female population need to be further explored.

In conclusion, changes in adolescent bone mass and composition in CAIS are largely unexplored, and this case series adds some insight into these processes. Although our results on lean mass are reassuring, our cases raise concerns that bone health is compromised. Given the small number of subjects, we cannot reach any conclusions about the benefits of early *vs.* late gonadectomy on bone health. However, our data do support an adverse effect of poorly treated hypogonadism on BMD. Given the rarity of the syndrome, our data call for multicenter, natural history studies to understand the risk of bone fragility and its most appropriate management in order to formulate HRT regimens that will maximize bone accrual and other health outcomes.

## Data Availability Statement

The raw data supporting the conclusions of this article will be made available by the authors, without undue reservation.

## Ethics Statement

The studies involving human participants were reviewed and approved by IRB of Children’s Hospital of Philadelphia. Written informed consent from the participants’ legal guardian/next of kin was not required to participate in this study in accordance with the national legislation and the institutional requirements.

## Author Contributions

AM collected the data and provided the first draft. MM assisted with data collection. LP, TK, and AK contributed in the critical review and editing of the manuscript. MV developed the final draft and assisted with data analysis. All authors contributed to the article and approved the submitted version.

## Conflict of Interest

The authors declare that the research was conducted in the absence of any commercial or financial relationships that could be construed as a potential conflict of interest.

## Publisher’s Note

All claims expressed in this article are solely those of the authors and do not necessarily represent those of their affiliated organizations, or those of the publisher, the editors and the reviewers. Any product that may be evaluated in this article, or claim that may be made by its manufacturer, is not guaranteed or endorsed by the publisher.
